# Gender distribution in authorship on research submissions at the European College of Veterinary Surgeons Annual Scientific Congress: 2012–2022

**DOI:** 10.1371/journal.pone.0343153

**Published:** 2026-03-04

**Authors:** Kathryn Pratschke, Kelly Blacklock, Alina Paczesna, Ishita Parakh, Jill R. MacKay, Fiona Mackay, Poppy Bristow

**Affiliations:** 1 Royal (Dick) School of Veterinary Studies, University of Edinburgh, Roslin, United Kingdom; 2 School of Social and Political Science, University of Edinburgh, Roslin, United Kingdom; 3 Veterinary Specialists Ireland, Clonmahon, Summerhill, Co. Meath, Ireland; University of Hafr Al-Batin, SAUDI ARABIA

## Abstract

The objective of this study was to explore gender distribution for authors on research presentations at European College of Veterinary Surgeons (ECVS) Annual Scientific Meetings from 2012–2022. Our populations for data collection included the ECVS Diplomate membership and authors listed for research submissions at the ECVS Annual Scientific Meetings between 2012–2022. Data was extracted from Conference Programs including year, first, second and senior (last) author names, and session type (scientific poster, short communication, resident forum). Authors were assigned a binary gender using a web-based algorithm to determine gender by first name. Gender demographics for ECVS Diploma holders between 1993–2023 was obtained from the ECVS Office for comparison to assess gender representation proportional to the specialty organisation, again this was based on a binary gender archetype. Although there are limitations to this approach, it is consistent with methodology in contemporaneously published papers in the human medical and veterinary fields. We identified 1353 research presentations, of which 1292 had complete information for all authors. At first author, men and women approached parity, but second and senior authors were more commonly men with the discrepancy being most marked at senior author level, and in the resident forum. If the first or senior author was a man, it was significantly more likely the second author would also be a man. There were no changes in authorship trends across the decade evaluated. In conclusion, women approached parity for first author but were under-represented as second and particularly senior author. In veterinary medicine, credit researchers receive is typically based on their position in the author list. First and senior author are more valued positions, with senior author usually having ownership of the project. Second author is the most valuable of the junior co-author positions but carries lower prestige than first or senior author. Further research is required to investigate underlying reasons contributing to ongoing gender disparity at senior author.

## Introduction

Over the past 10–15 years numerous publications have documented systemic gender bias impeding equity and career progression for women working in human healthcare and various medical specialty fields [1–19]. These concerns have been particularly prominent in disciplines traditionally viewed as masculine, such as surgery [2–6]. The term gender is generally understood to mean social constructs of men/male, women/female and gender-diverse individuals as opposed to biological sex (male, female) which relates to the chromosomes with which one is born. As a social construct, gender does not have a single fixed definition. It can vary between different societies, can change over time, and has significant intersection with other markers of inequality such as ethnicity, age, sexual orientation and socioeconomic status [20].

As a profession, veterinary medicine was historically dominated by men, however broadly equal numbers of undergraduates were men and women by the late 1980s [21]. By the mid-2010s, women comprised over 70% of new veterinary graduates in the UK and US [22]. Between 2010 and 2019 approximately 75% of graduating veterinarians in the UK were women, and in 2021 approximately 80% of graduating veterinarians in the USA were women [23,24].

Despite this progressive feminization of the profession since the late 1980’s, women in veterinary medicine remain under-represented in senior management and leadership positions [25,26] and they are promoted at slower and lower rates than their male peers [8,27,28]. A significant gender pay gap persists [28–30] and they receive fewer professional honours [8,21,25–28,30–32]. This resembles the ‘leaky pipeline’ phenomenon described within Science Technology Engineering Mathematics and Medicine (STEMM) subjects in 1996, whereby women are consistently under-represented at higher levels despite equal representation at the recruitment stage [31,32]. The term ‘leaky pipeline’ suggests a passive loss of talent, but the reality is frequently that women are lost from these professions due to structural issues and barriers that impede progression [8,24,28,31,33,34]. There are interesting comparisons to be made between the ‘leaky pipeline’ idea and the Matthew effect first reported by Robert Newman in 1968 and subsequent Matilda effect described by Margaret Rossiter [35,36]^.^ The Matthew effect describes the psychosocial phenomenon whereby scientists that are already well known tend to gain greater acclaim and recognition from collaborations than do their more junior counterparts, even where contributions are equal, or the junior scientist has contributed more to the work. Ironically, Newman’s paper was based on work performed largely by Harriet Zuckerman, who Newman later admitted should have been named as co-author [36]. This concept was expanded to the Matilda Effect in 1993 by Margaret Rossiter, in a paper discussing systematic under-recognition of the achievements and contributions of women in science; the eponymous Matilda was Matilda Joslyn Gage, an American feminist, suffragist and sociologist in the nineteenth century [36].

Numerous publications document systematic under-representation of women as invited conference speakers in a range of human healthcare specialty fields [3,4,7,9,37–54]. Similar findings have been recently reported for the speciality of veterinary surgery [55]. Several publications in human healthcare specialty fields have also evaluated gender distribution for authorship on research abstracts and posters at specialty conferences, but we currently lack comparable information for veterinary specialty meetings [7,39,42,43,45,50–52,56]. One recent veterinary paper reviewed publication rate of abstracts from the Veterinary Endoscopy Society annual meetings between 2004–2019. Although the authors did not delve into the details or distribution of author genders, they found that male first authors outnumbered female, but abstracts with a female first author were twice as likely to be subsequently published as a full paper [57].

Our objective was to explore gender distribution at first, second and senior authorship on research submissions presented at European College of Veterinary Surgeons (ECVS) Annual Scientific Meetings between 2012–2022, including identification of gendered patterns of collaboration. The secondary objective was to compare gender distribution for senior authors on research submissions against ECVS Diplomate demographics from 1993–2023, and with invited speaker demographics for the same meetings [55].

## Materials and methods

The institutional Human Ethical Review Committee (Ref: HERC 23_056) approved this project. Ethical approval was granted for research investigating gender distribution across authorship on research submissions presented at European College of Veterinary Surgery Annual Scientific Meetings (2012–2022), with the Committee providing a favourable ethical opinion. The Ethics Review Committee waived a need for the study to gain specific consent from individuals listed as authors on the Conference Programs on the basis that we adhered to the ethics guidelines of the Association of Internet Researchers when mining for additional data from people on the list (to assign gender), and we used only data which publicly available on veterinary based websites. Our first data source was the ECVS Diplomates list as of May 25th, 2023, provided in anonymised format by the ECVS Secretarial Office to give only Diplomate gender and year of passing the ECVS Diploma examination. Our second data source was the Conference Programs. Data recorded from Conference Programs for ECVS Annual Scientific Meetings from 2012–2022 included first and last name of the first author, second author, and senior author for all blind reviewed research submissions. We also recorded the type of presentation (short communication, scientific poster, or resident forum presentation). If an individual was not identifiable in the European Board of Veterinary Specialization (EBVS) online searchable database or another Specialty College website, the name was searched in Google to identify individuals via institutional websites, LinkedIn profiles, journal publications, and social media. If it was not possible to identify a first name and therefore gender for any of the authors on a research submission, this submission was excluded from further analysis. Senior author data were bench-marked against the demographics of ECVS Diplomate membership as of May 2023 (meaning individuals who have successfully passed the certifying examination to achieve an ECVS Diploma). Senior author data were also compared to data for invited speakers at the same Scientific Meetings, to gauge the impact that a blind review process might have on gendered involvement when compared to data available for invited speakers at the same meetings [55]

### Gendering authors

Following methodology described in previous studies [45–50], we used a web-based algorithm to determine gender by first name (https://gender-api.com). Gender-API has been compared favourably to other gender detection tools including NamSor (https://namsor.app/), genderize.io (https://genderize.io/), Wiki-Gendersort (https://github.com/nicolasberube/Wiki-Gendersort), and is one of the most accurate name-to-gender inference services [58–60]. Probabilistic name-to-gender inference services have been widely used in politics and marketing as well as research. They rely on huge, regularly maintained databases of names drawn from publicly available sources such as government records. The Gender-API database evaluates a name and assigns a binary gender category (man or woman) with an accuracy rating from 0–100 based on the number of records in the training database for each name. We used a 70% threshold for acceptably reliable accuracy, consistent with previous studies [61,62]. Any name with a value less than or equal to 70% was manually reviewed to confirm gender and any preferred pronouns indicated, based on an online professional profile. If gender could not be assigned for any author, we excluded the associated research submission from further analysis. We used binary gender archetypes (man/male or woman/female) based on the general paucity of research into gender equity in veterinary surgery and because it is consistent with the available relevant literature thereby allowing comparisons to be made. There were also practical restraints in terms of time, availability and accessibility of data and allocation of resources. Data protection laws in our country and ethical review stipulations meant we could not directly contact individuals to clarify their gender identity. We did not divide the individuals in our data set into male or female biological sex based on first name, as this would have meant assuming an individual with a ‘woman’s’ name must be biologically female, and someone with a ‘man’s name’ must be biologically male. We did not believe these would be acceptable assumptions.

### Statistical analysis

Statistical analysis was performed using a commercially available statistical software package (IBM SPSS, IBM Corporation, Chicago, IL, USA). Descriptive statistics were computed for all variables. Data were identified non-normal by means of Kolmogorov-Smirnov (p < 0.001) and Shapiro-Wilk (p < 0.001) tests, so values are reported as median (range). Pearson Chi-Square Test for Association was used to identify significant relationships between different author positions, gender, year, and type of presentation. Where significant relationships were confirmed, these variables were tested separately via binary logistic and forward logistic regression, with results reported as odds ratios (OR) with 95% confidence intervals (CI), and associated P values.

## Results

### ECVS membership

**Fig 1:**
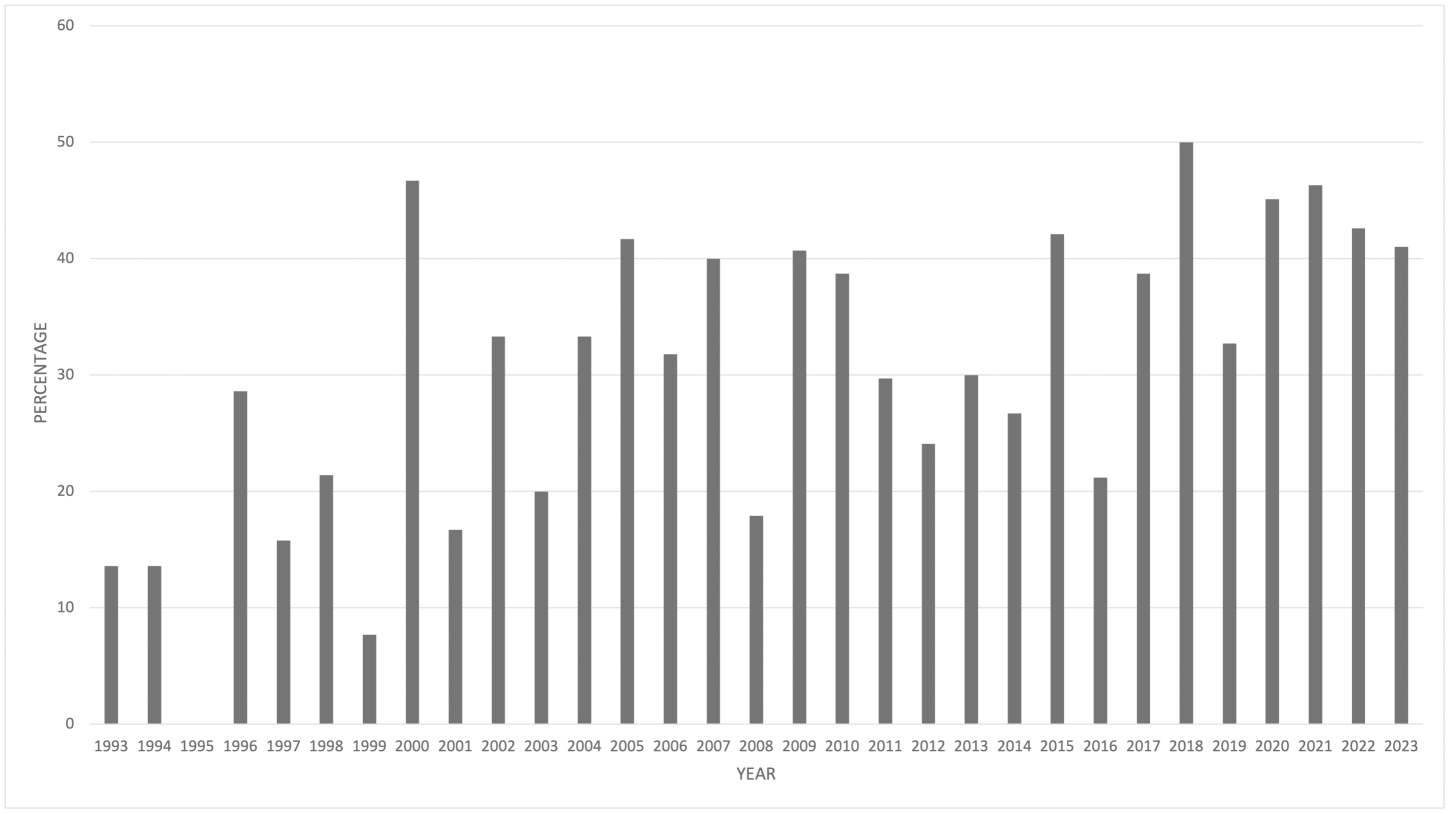
Percentage of new ECVS Diplomates each year that were female, from 1993-2023. This figure shows the proportion of new ECVS Diplomates who were women each year between 1993-2023. There has been a steady but sometimes inconsistent increase in the proportion of women becoming Diplomates, with the figure reaching 33.8% overall in 2023.

Three hundred and twelve (33.8%, 312/924) ECVS Diplomates registered between 1993–2023 were women. In 1993, when the College was founded, 6/44 (13.6%) of Diplomates were women. In 2002, a decade before the meetings we evaluated, 19.7% of ECVS Diplomates were women. (Fig 1)

### Research presentations between 2012–2022 (Table 1)

Between 2012–2022, 1353 research presentations were selected by ECVS Programme Committees via a process of blind review. Our analysis of the subsequently published programmes identified 1292 presentations that had complete information for gender of all authors. Any presentations that did not have complete information were excluded from analysis. This resulted in 4.5% of submissions being excluded, which we believe was sufficiently low as to not compromise the findings.

**Table 1:** Author gender distribution for research submissions.

Scientific posters	2012	2013	2014	2015	2016	2017	2018	2019	2021	2022
First Author Men %	56.3	59.7	55	68.4	55.3	56.9	37	49.1	48.9	57.1
First Author Women %	43.7	40.3	45	31.6	44.7	43.1	63	50.9	51.1	42.9
Second Author Men %	66.6	66	53.2	62.8	64.7	61.5	66.6	57.7	56.1	34.3
Second Author Women %	33.4	34	46.8	37.2	35.3	38.5	33.4	42.3	43.9	65.7
Senior Author Men %	73.5	66.1	63.8	54.4	57.5	53.2	73.1	76.5	70.8	58.1
Senior Author Women %	26.5	33.9	36.2	45.6	42.5	46.8	26.9	23.5	29.2	41.9
SHORT COMMUNICATIONS										
First Author Men %	86.1	74.4	57.1	39.7	55.2	55.3	49.1	49.1	56.9	47.2
First Author Women %	13.9	25.6	42.9	60.3	44.8	44.7	50.9	50.9	43.1	52.8
Second Author Men %	61.5	47.2	46.2	42.6	65.3	53.5	62.5	48.8	59.2	46.3
Second Author Women %	38.5	52.8	53.8	57.4	34.7	46.5	37.5	51.2	40.8	53.7
Senior Author Men %	72.7	75.7	71.4	61.3	71.4	59.1	65.4	65.3	70.1	60
Senior Author Women %	27.3	24.3	28.6	38.7	28.6	40.9	34.6	34.7	29.9	40
RESIDENT FORUM										
First Author Men %	65	40	62.1	58.6	51.9	44.4	34.5	53.3	53.3	60
First Author Women %	35	60	37.9	41.4	48.1	55.6	65.5	46.7	46.7	40
Second Author Men %	72.2	61.1	69	77.8	76	80	50	69.2	77.8	44
Second Author Women %	27.8	38.9	31	22.2	24	20	50	30.8	22.2	56
Senior Author Men %	65	65	79.3	86.2	51.9	66.7	72.4	76.7	73.3	73.3
Senior Author Women %	35	35	20.7	13.8	48.1	33.3	27.6	23.3	26.7	26.7

Percentage of men and women as first author, second author and senior author for 1292 research presentations at ECVS Annual Scientific Meetings between 2012–2022. Results are presented within three categories, scientific posters, short communications and resident forum.

**Fig 2:**
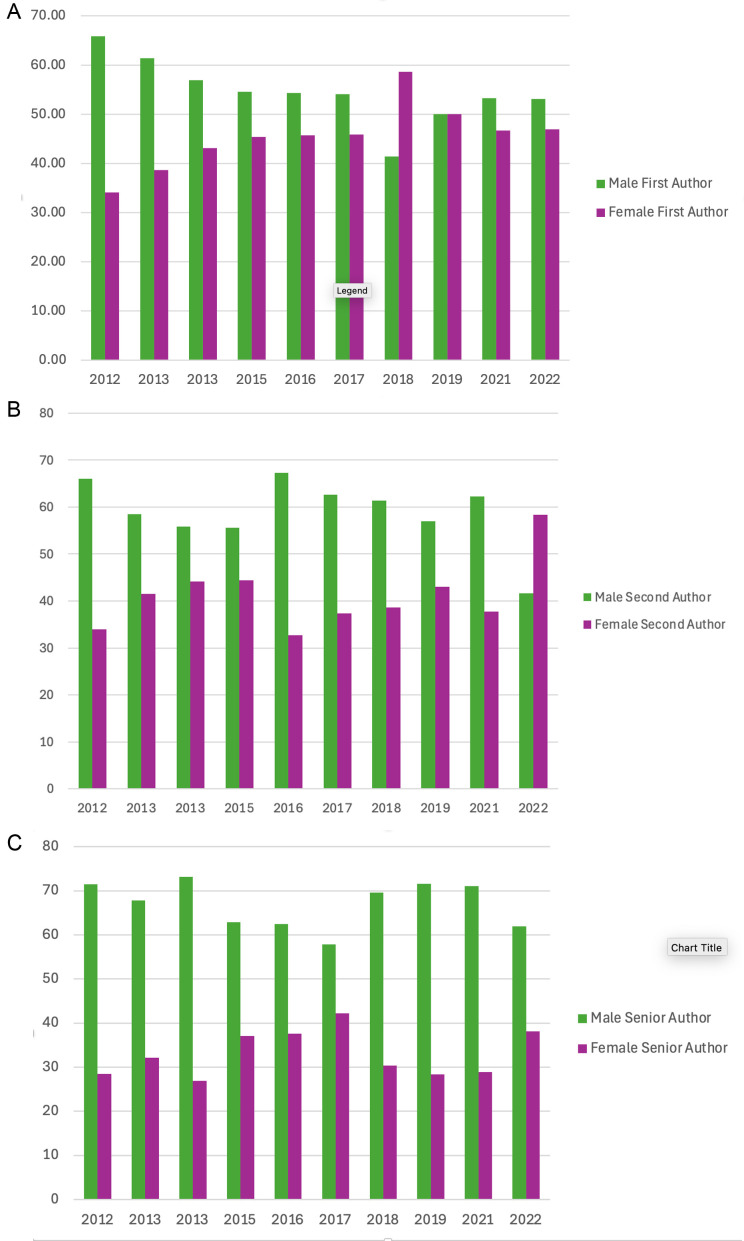
a-c. Gender distribution at first, second and senior author on research submissions. These bar charts illustrate gender distribution (male or female) at first author (2a), second author (2b) and senior author (2c) level across 1292 research submissions presented at ECVS Annual Scientific meetings between 2012–2022. The greatest discrepancy was at senior author in all types of research presentation.

There were first and last authors only with no second author listed for 82 scientific posters, 78 short communications and 23 resident forum presentations. Across all research submissions included for data analysis, at first author men and women were reasonably close to parity with men having a median of 55.3% (range 34.5–86.1%) compared to 44.75% (range 13.9–65.5%) for women. At second author, men had greater involvement at a median of 61.5% (range 34.3–80%) compared to 38.5% (range 20–65.7%) for women. Senior authors were more commonly men, with a median of 68.4% (range 51.9–86.2%) compared to a median of 32.7% (range 13.8–48.1%) for women (Fig 2a-2c).

When we looked at scientific posters only, men accounted for a median of 56.6% (range 37–68.4%) first author positions, and women 44.2% (range 31.6–63%). Noticeably fewer second authors were women however, with a median of 37.9% (range 33.4–66.6%) compared to 62.2% (range 34.2–66.6%) for men. At senior author the picture was similar with women having a median of 35% (range 23.5–46.8%) compared to 65% (range 53.2–76.5%) for men.

In the short communications sessions, men accounted for a median of 55.3% (39.7–86.1%) first author positions, and women 44.7% (range 13.9–60.3%). There were similar findings for second author, with a median of 51.2% (range 42.6–65.3%) for men and 48.9% (range 34.7–57.4%) for women. The largest discrepancy was at senior author where women dropped to a median of 32.3% (range 24.3–40.9%) compared to median 67.8% (range 59.1–75.7%) for men.

**Fig 3:**
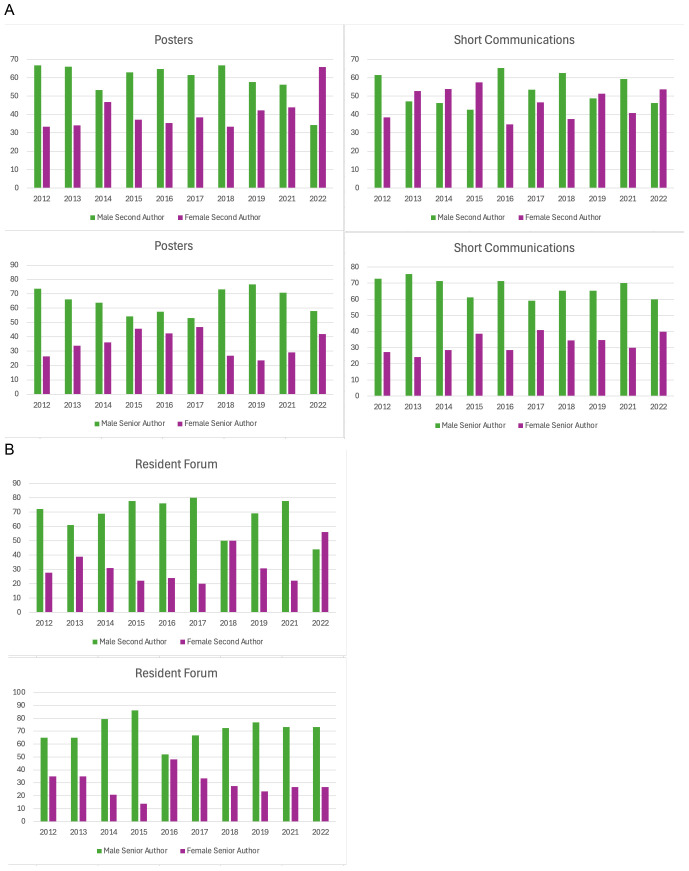
Gender distribution at second and senior author in poster and short communications. **a.** This bar chart illustrates the gender distribution (male or female) at second and senior author level in the poster and short communication sessions presented at ECVS Annual Scientific Meetings 2012-2022. **b.** This bar chart illustrates the gender distribution for second and senior author at the resident forum at ECVS Annual Scientific meetings between 2012-2022. The greatest discrepancy between men and women at senior author level was seen in the resident forum.

In the resident forum, men and women were comparably represented at first author, with a median of 53.3% (range 34.5–65%) for men and 46.7% (range 35–65.55) for women. At second author however men accounted for a median of 70.7% (range 44–80%) compared to 29.3% (range 20–56%) for women. The same was true at senior author, with a median of 72.8% (range 51.9–86.2%) for men compared to 27.2% (range 13.8–48.1%) for women. The difference between men and women at second and senior author levels was more pronounced in the resident forum than any other presentation type (Fig 3a and 3b).

The number of ECVS Diploma holders that are women has steadily increased over time. For example, during the 10 years covered by this study it went from 27% to 33.2%. To see if this trend for increasing involvement of women at Diplomate level was reflected in the numbers of women at senior author, we compared the first 5 years conference data (2012–2016) to the second 5 years (2017–2022). However, we found that representation of women at senior author decreased for scientific posters (36.9% to 33.6%) and resident forum presentations (30.5% to 27%) although it increased for short communications (29.5% to 36%). The median percentage senior author positions occupied by men increased across the decade for all types of research submission (scientific posters 63% to 66.3%, short communications 65.3% to 71.4%, resident forum 70% to 72.4%).

### Gendered patterns in collaboration on research submissions

Across all research presentations, submissions where first and senior authors were both men accounted for 36.2%. Those with a woman at first author and man at senior author accounted for 34%. Presentations with women at both first and senior author accounted for 15% and those with a man at first author and woman at senior author for 14%. Interestingly, these patterns of gendered collaboration reflect what has previously been reported for research abstracts submitted to both endocrine surgery and academic surgery conferences in human healthcare [50,52]. Pearson Chi-Square Test for Association identified no significant relationship between the gender of first and senior authors for research submissions (p < 0.116). However, there was a significant association between gender of first and second authors (p = 0.016) with binary logistic regression including Hosmer and Lemeshow test for goodness of fit identifying 35% increased odds the second author would be male if the first author was male (OR 1.35 (95% CI 1.1–1.7, p < 0.001). Pearson Chi Square Test for Association also found a significant relationship between gender of the senior author and second author (p = 0.008), with binary logistic regression including Hosmer and Lemeshow test for goodness of fit suggesting 39% increased odds the second author would be a man when the senior author was male (OR 1.39 (95% CI 1.1–1.78, p = 0.01).

Forward stepwise logistic regression was used to assess variables potentially impacting first author and senior author genders. Presentation type, gender of other authors and Diplomate status of the senior author had no effect on first author gender, but the odds the first author would be a man increased by 5.4% (OR 1.054, 95% CI 1.014–1.095, p = 0.007) with every year that passed from 2012−2022. Year, type of presentation and first author gender had no effect on senior author gender, however Diplomate status was significant. If the senior author was a Diplomate, the odds they were a man increased by 43% (OR 1.436, 95% CI 1.090–1.893, p = 0.01).

In summary, relating to our primary objective we found that at first author, men and women approached parity, but second and senior authors were more commonly men with the discrepancy being most marked at senior author level, and in the resident forum. Our secondary objective to evaluate gendered patterns or collaboration showed that when the first or senior author was a man, it was significantly more likely the second author would also be a man. No comparable beneficial pattern was identified for women. Representation for women at senior author and within ECVS Diplomate membership, were both higher than the percentage invited speakers that were women at the same conferences.

## Discussion

Overall, gender distribution at first author was broadly comparable in research submissions for ECVS Annual Scientific Meetings between 2012–2022. At second author, the same was true for short communications, but involvement of women at second author was lower for scientific posters (37.9%) and particularly for the resident forum (29.3%). At senior author, a median of 32.3% senior authors were women, with the greatest involvement being on scientific posters (35%) and the lowest in resident forum presentations (27.2%). These numbers for senior author approach or equal proportional representation when compared to the ECVS Diplomate membership, which was 33.8% in 2023. We previously reported that women were consistently under-represented as invited speakers for ECVS meetings between 2012–2022 compared to Diplomate demographics (21% versus 33.8%) [55]. When a research presentation such as a short communication, scientific poster, or resident forum presentation is submitted, it goes through a process of blind review. Name, gender, nationality, and place of work are unknown to the reviewers and acceptance is based therefore only on merit. Our data suggests that this blind review process increases involvement of women at all author levels when compared to invited speaker demographics. This phenomenon, that women have increased involvement when presentations go through blind review compared to when there is a direct invitation, has been previously reported for research submissions compared to speaker invitations at conferences in colorectal surgery, evolutionary biology, paediatric medicine and neurosurgery in human healthcare specialties [7,42,43,45]. Our data also shows that every year from 2012–2022, the odds a first author on a research submission would be a man increased by 5.4%. This runs counter to the steady increase in numbers of women undertaking residency training and achieving Diplomate status over the past 20 years and is difficult to explain.

We found that second author gender on research presentations was significantly influenced by gender of both first and senior authors. Men were more likely to benefit from second authorship if the first and/or senior authors were men. There was no comparable benefit seen for women when first and/or senior authors were women. The UK Research Integrity Office suggests that in STEMM fields co-authors should have significantly contributed to conception of the project and/or performance of relevant research as well as being involved with finalising work for publication [63]. It’s common practice in scientific publications to order the authors in a manner that reflects their respective contributions, with the last author position typically reflecting the senior and more established contributor, often with ownership of the research topic or project. Invitations from an established researcher to a more junior colleague are a valuable way to advance the junior colleagues’ career, build a research network, and increase visibility. That men in senior positions are more likely to engage in research collaborations with other men has been previously documented, with our findings providing further confirmation of the phenomenon in veterinary surgery [4,27,50,52,64,65]. Similar bias does not however exist where women are senior collaborators, either in the published literature or in our data, suggesting that men benefit from gender concordance, but women do not [27,52,65].

While it is encouraging to see figures reflecting proportionate representation compared to ECVS Diplomate demographics at senior author for some presentation types, it is disappointing that women’s involvement at senior author overall trended downwards across the decade on scientific posters and resident forum sessions. Additionally, the lowest figures for women at senior author were in the resident forum, at only 27.2%. Senior author on resident forum submissions is most commonly the main ECVS resident supervisor. It may sometimes be another senior surgeon involved in their training, one with a significant research program in the residents training institution. Given the importance of women as educators and strong role models for retention of more junior women training within STEMM fields, these facts may be a cause for concern given that they suggest a relative under-involvement of women in senior roles working with residents in training [15,66]. As more women become Diplomates and develop their careers, we might logically expect to see increasing involvement of women at senior levels leading clinical research in our field, but this is not what our data suggests.

It is important therefore to consider what factors might positively and/or negatively influence involvement of women in research in veterinary surgery,. Men often have greater self-confidence and self-belief, and they are more likely to actively seek involvement in collaborations while women tend to under-estimate and under-value their own achievements and abilities [18,67–69]. Many women are also aware of the negative connotations of exhibiting agentic characteristics typically associated with men, such as assertiveness, self-confidence, and directness [68,70]. It is still widely considered important, albeit often at an unconscious level, that women be likeable, collegial, friendly and caring; they are often judged negatively for failing to meet these stereotypes [15,18,68,71–74]. We suggest it is important those in a position to offer mentorship, or invite junior colleagues to join research collaborations, consider whether they are inadvertently only rewarding those with extrovert tendencies and greater self-confidence. We believe it would also be beneficial for those in senior positions to be aware of the possibility of habitual gender homogeneity in their collaborations. It may be helpful if veterinary specialty conferences and groups adopt pro-active stances to encourage women in their specialty to become research active, for example identifying appropriate mentors to work with those who are less established in research. The ECVS could also consider setting up a working group such as the Association of Surgeons of Great Britain and Ireland women in surgery working group, to help forward gender equity [56,75]. To be clear, we are not advocating for enforced quotas. Diversity of inclusion does not mean compromising on excellence and implicit in any argument for gender equity is that no group is disadvantaged. Rather, it means ensuring that everyone has an equal opportunity to progress their careers regardless of their gender, instead of progress being influenced by a continued default to habitual practices.

In academic medicine, women remain concentrated in lower ranked positions, receive lower salaries, and take longer to be promoted despite having equivalent qualifications and experience [2,12,46,76]. Research suggests that in STEMM fields women are judged more harshly in peer-reviewed grant applications, receive smaller sums of money, are less likely to be perceived as leaders, and have lower success rates on re-application [10–12,77]. A similar picture has been described for academic veterinary medicine [8,25,27,28,78,79]. A study reporting gender differences in research collaborations and career advancement in veterinary faculty in the USA found that even controlling for relevant factors men had 43% higher odds of attaining a more advanced academic rank than did women [27]. A study from 2021 that assessed gender distribution in academia at a global level found that while women occupied between 45.5% and 47.9% of academic positions, they were clustered in low ranked positions [25]. Implicit gender bias is a key player in perpetuating such gender inequity, and one that is often misunderstood. Critical to understanding implicit gender bias is that no-one is exempt from it – it’s something we are all exposed to through our upbringing and daily lives. Implicit bias is usually based on years of exposure to cultural messages, social norms and expectations, and is frequently different to consciously held opinions [18,30,68]. Storage and co-workers (2020) surveyed 3618 individuals, finding evidence of consistent implicit bias for a gender-brilliance stereotype favouring men over women, i.e., man = brilliance [80]. This was present in children as young as 8 years old suggesting this bias is pervasive, widespread, and fostered in early life. Research in human healthcare documents implicit bias in surgical residency training, manifesting through communication from other doctors, other residents and patients as well as in formal resident assessments and references [14,18,19,74,81]. This reinforces the importance of ongoing education to improve general understanding and awareness of implicit bias and how it impacts a significant proportion of the workforce.

Other barriers to women’s involvement in clinical research that could help career progression include the challenges associated with pregnancy, family and childcare. There is a wealth of evidence from human healthcare showing these are significant obstacles for women trying to develop surgical careers [15,56,75,82,83]. A veterinary study from 2025 reported that female veterinarians were disproportionately affected during the COVID-19 pandemic in terms of childcare responsibilities, professional standing and mental health [84]. Similarly, a disproportionate impact on working life due to childcare requirements has been reported for female veterinary specialists in both surgery and internal medicine [85,86]. The relative paucity of strong, inspiring and supportive mentorship for female surgical trainees is well recognised as contributing to loss of women from surgical professions and it is likely to be the same for veterinary surgical trainees [19,56,66,67,82,87–90].

The retrospective nature of our study means we considered gender but could not assess the impact of other important demographic indicators of diversity including race, socioeconomic status, religion and sexual orientation at this time.. We recognise that binary gender has limitations, but we used this based on the general paucity of research into gender equity in veterinary surgery, and also because it is consistent with the available relevant literature [8,24–29,69,78]. There were also practical issues with availability and accessibility of the necessary information. Accrual of granular self-reported demographic data is not currently standard within veterinary specialty colleges and veterinary organisations in Europe, and this would be required for more detailed assessment We used Gender-API to allocate binary gender to our data set of speakers, with a 70% cut-off for accuracy as previously reported [62]. Gender API gives accessibility, high accuracy for most names, and can be used on large datasets with high processing speed. Weaknesses include difficulty assigning gender and accuracy below 70% for some names (often non-English or non-Western names).

In conclusion, our data shows that broadly equivalent numbers of men and women were first author on research submissions from 2012–2022. The same was true for second author on scientific posters but men comprised >60% of second authors on other presentation types. Women were close to proportionate representation compared to ECVS Diplomate membership at senior author for research presentations other than in the resident forum where they were under-represented at 27.2%. We also identified gendered patterns of collaboration that we suggest primarily benefits men, and we have discussed some of the potential reasons why women remain under-represented at more senior levels. We believe our findings highlight the importance of recognising and discussing barriers that may currently exist for women wishing to progress their academic careers in the speciality of veterinary surgery.
